# Quality of Reports on Drug Toxicity in Eudravigilance: A Safety Physician’s Perspective

**DOI:** 10.1007/s43441-025-00884-x

**Published:** 2025-10-16

**Authors:** Jana Brajdih Čendak

**Affiliations:** Billev Pharma East Ltd, Ljubljana, Slovenia

## Abstract

**Background:**

European legislation requires Marketing Authorization Holders (MAHs) to continuously monitor Eudravigilance (EV) data and inform the European Medicines Agency and national competent authorities of validated safety signals. The process follows the Good Pharmacovigilance Practice Module IX and is based on the review of individual case safety reports (ICSR) both from the MAH’s internal database and from EV. The data is reviewed and evaluated by a medically trained person, who should, based on the information provided in the case, determine the causal relationship between the suspect drug and the reported event. In order to do this with a certain degree of confidence, the case needs to report enough high-quality information on the drug, patient and adverse event, without significant confounders.

**Methods:**

For this purpose, we performed an evaluation of data quality of ICSRs within the Eudravigilance database, focusing on drug toxicity cases for five commonly implicated substances: paracetamol, diazepam, fentanyl, quetiapine, and fluoxetine. A medically driven review was conducted on 500 randomly selected ICSRs from 2015 to 2024. A detailed quality assessment framework was developed and applied, scoring cases across several criteria (including data on suspect drugs, patient demographics, adverse event description, time to event, and case narratives), resulting in a maximum quality score of 25.

**Results:**

Main study findings revealed a generally low data quality, with an average score of 11.57 out of 25. Key quality deficiencies included improper classification of drugs as suspects (e.g., reporting concomitant medications or treatments as suspects), reporting of underlying diseases and indications as adverse events, lack of information on patients’ medical history and missing time-to-event information. Cases from non-European Economic Area (non-EEA) countries and consumer-reported cases exhibited the lowest quality, while regulatory agency-reported cases were of higher quality. The study also identified a frequent misclassification of non-prescription or illicit substances (e.g., fentanyl) as prescription products, complicating signal detection and causality assessments. The analysis highlights a very important gap in pharmacovigilance signal detection and evaluation processes, underscoring risks for misleading results, increased workload, and potential misinterpretation of product safety profiles. The results highlight the need for enhanced case reporting trainings, improved quality control, better follow-up processes, and a collective mindset shift across stakeholders to prioritize data quality.

**Conclusion:**

In conclusion, significant improvements in the completeness, accuracy, and clinical relevance of ICSRs are essential to support effective safety signal detection and benefit-risk assessment in the post-marketing surveillance of medicinal products.

## Introduction

Eudravigilance (EV) is a system for managing and analyzing safety information on medicinal products, developed and maintained by the European Medicines Agency (EMA). All Marketing Authorization Holders (MAHs) in the European Economic Area (EEA) are obliged to report adverse events related to their authorized medicines into the system.

Commission Implementing Regulation (EU) 2025/1466 [1] requires MAHs to continuously monitor Eudravigilance data and inform EMA and national competent authorities of validated signals detected during the process.

Following a pilot period that started on 22 February 2018 and only mandated the use of Eudravigilance data for a limited list of active substances and combinations, the new Implementing Regulation (EU) 2025/1466 now require all MAHs with medicinal products authorized in the EEA shall monitor the data available in the Eudravigilance database and use it as an additional source of safety information to support their processes and enhance signals detected through other sources.

Signal detection is a data review process, that is intended to detect potential safety issues, related to a particular product in the post-marketing phase. The process follows the Good Pharmacovigilance Practice (GVP) Module IX: Signal Management [2] and is based on the review of individual case safety reports (ICSR) both from the MAH’s internal database and from EudraVigilance. The data are reviewed and evaluated by a medically trained person (safety physician), who should, based on the information provided in the case, determine the causal relationship between the suspect drug and the reported event. To do this with a certain degree of confidence, the case needs to report enough high-quality information on the drug, patient and adverse event, without considerable confounders.

The causal relationship determination considers the drug pharmacology, known safety profile, the patient’s age, sex, pathology and medical history and the temporal relationship between the administration of the medicine and occurrence of the event.

In our over 15 years long experience with signal detection and using Eudravigilance data since the start of the pilot phase in 2018, we have repeatedly seen that many ICSRs lack such critical information and that they are often practically useless, if not misleading, when performing signal detection activities.

For this reason, we have performed an evaluation of data quality of ICSRs, as reported in EudraVigilance, for a specific subset of substances, which are, in our experience, especially difficult to adequately medically assess and often lack crucial information: cases of drug toxicity.

## Methods

### Data Acquisition

A review was conducted of cases of poisoning and toxicity, reported in EV on 5 medicinal products, that are commonly implicated in intoxications, both intentional and accidental [3–6]:

1. Paracetamol.
2. Benzodiazepines (diazepam).
3. Opiates (fentanyl).
4. Antipsychotics (quetiapine).
5. Antidepressants (fluoxetine)^1^

Eudravigilance cases were obtained via line listing from the Eudravigilance Data Warehouse (EVDAS). The following criteria were used to retrieve data:

*Active substance (high level)*

*MedDRA HLT poisoning and toxicity*

*Period: 01/01/2015–31/12/2024 (10 years)*

100 cases were randomly selected from the obtained line listings for each substance using the RAND() function in MS Excel. This function randomly assigns a number to each row in the worksheet. By sorting the numbers from largest to smallest (or vice versa), a random selection is obtained.

A Level 1 (public) CIOMS form for each individual case was downloaded.

### Quality Evaluation

The cases were reviewed individually, according to the below listed criteria. The review was performed by a trained physician with over 15 years of experience as an emergency medicine physician and a medical advisor for pharmacovigilance.

As part of the quality evaluation, the following criteria were considered:

1. **Data on substance**: Adequacy of reported pharmaceutical form, dose and dosing regimen are adequately reported and allow for an unambiguous identification of what, how and how much substance the patient took. Drugs used to treat the AE are not reported as suspect. Concomitant drugs are not reported as suspect (quality of data).
2. **Intoxication type**: It is clearly stated whether the case involved an intentional or accidental overdose, or toxicity at therapeutic doses.
3. **Time to event**: Number of doses administered before toxicity are reported or can be deduced from the reported timelines (dosing regimen and days to event reported).
4. **Adverse reaction**: The reported PTs (preferred term according to MedDRA coding system) are coherent, medically correct and plausibly related to the reported intoxication or toxicity syndrome. Underlying conditions are not reported as Reaction List PTs. Indications for use are not reported as Reaction List PTs (quality of data).
5. **Patient**: Age or age group, sex, medical history, indication for drug use (if applicable) are clearly stated.
6. It is clear from the case which is the **main suspect drug(s)** yes/no.
7. **Concomitant substances**: If reported, it is clearly defined which substances are co-suspect and which concomitant. Drugs used to treat the AE are not reported as concomitant. If there are no concomitant substances, this is clear from the narrative and/or other references (quality of data).
8. **Seriousness criteria**: Correctly reported yes or no.
9. **Quality of additional information**: Additional data that allows for a comprehensive medical evaluation (e.g. case narrative, sender comment, data on lab results, follow-ups, links to literature references…).

0—None available; 1—no useful data provided; 2—limited usability of additional data, assessment; 3—limited medical information provided, difficult to assess the actual course of events, but it can be deducted in general what happened to the patient; 4—most important medical information provided, time course clear. Clearly determined what happened to the patient; 5—comprehensive medical information described with clear time course of the events and additional information (e.g. follow-up data, data on clinical investigations…).

The summary of the scoring system and criteria is presented in Table 1.

**Table 1:** Criteria and scoring system in the evaluation of cases quality

	Criterion	Scoring
1	**Data on suspect drug(s)**	**0–5**
	Pharmaceutical form	0 or 1
	Dose/dose regimen	0 or 1
	Quality of data	0–3
2	**Intoxication type**	**0 or 1**
3	**Time to event**	**0 or 1**
4	**Adverse reaction (s)**	**0–4**
	Adequacy of reported AEs	0 or 1
	Quality of data	0–3
5	**Patient data**	**0–4**
	Age/age group	0 or 1
	Sex	0 or 1
	Medical history	0 or 1
	Indication	0 or 1
6	**Main suspect drug(s) reported**	**0 or 1**
7	**Concomitant drugs**	**0–3**
	Adequately reported	0 or 1
	Quality of data	0–2
8	**Seriousness adequately reported**	**0 or 1**
9	**Additional info quality**	**0–5**
	**Total score**	**0–25**

Maximal score: 25

Based on the total score, the following quality classes were defined and assigned to each case (Table 2):

**Table 2:** Quality classes for cases evaluation

Quality	Score	Description
Very low	0–5	The case reports only basic data and/or contains many confounders. The data is not comprehensively reported. Determination of causality of a specific drug-event combination is not possible
Low	6–10	The case reports only basic data. Confounders are limited, but assessing causality of the specific drug-event combination is only tentatively possible
Medium	11–15	The case reports sufficient, but low-quality data that allow for assessing causality of the specific drug-event combination
High	16–20	The case contains good quality medical data that allow for assessing causality of the specific drug-event combination
Very high	21–25	The case contains comprehensive, high-quality medical data that can be used for assessing causality of the specific drug-event combination and brings added value for signal detection and benefit-risk evaluation of the product

Additionally, where possible, the outcome of the case was recorded (fatal yes or no) and the causality was re-evaluated based on available data in the CIOMS form. These two criteria were however not considered for quality evaluation, as they were not available for all cases. This information was used in the discussion on the possible causes of quality issues.

### Statistical Analysis

Statistical analyses were conducted using Python version 3.11 with several libraries and run as a Google Colab notebook to generate results and figures.

For each substance, the number of cases (N), mean, standard deviation (SD), median, first quartile (Q1), third quartile (Q3), and interquartile range (IQR) were calculated. Differences in mean total quality scores between substances were assessed using a one-way analysis of variance (ANOVA), followed by Tukey’s Honest Significant Difference (HSD) test for pairwise comparisons. Considering the possibility of a non-normal distribution, a Kruskal–Wallis H test was also performed as a nonparametric alternative. ANOVA assumptions were evaluated using the Shapiro–Wilk and D’Agostino–Pearson tests (for normality of residuals) and Levene’s and Bartlett’s tests (for homogeneity of variances).

To evaluate normality of distribution of quality scores across the scoring range (0–25), a Chi-square goodness-of-fit test compared the observed score frequencies to those expected under a discrete uniform distribution. This was done for the overall dataset and for each substance separately.

Boxplots were generated to display score distributions per substance, and bar plots with 95% confidence intervals illustrated mean differences.

## Results

Overall, the number of cases retrieved from Eudravigilance for a period of 10 years in presented in Table 3, while Table 4 shows the cases distribution across years. The distribution was not equal, but all years were represented in the dataset.

**Table 3:** Number of cases retrieved from eudravigilance per substance

Paracetamol	6551
Diazepam	2656
Fentanyl	2678
Quetiapine	2888
Fluoxetine	1054

**Table 4:** Distribution of retrieved cases by year

	Paracetamol	Diazepam	Fentanyl	Fluoxetine	Quetiapine
2024	18	16	21	12	12
2023	15	16	11	10	12
2022	6	9	5	8	10
2021	9	6	7	9	13
2020	8	10	6	8	16
2019	10	4	5	9	4
2018	9	15	7	12	4
2017	14	12	23	18	16
2016	4	4	8	2	2
2015	7	8	7	12	11

### Overall Evaluation of Cases Quality

Overall, the quality of cases of drug toxicity, reported in Eudravigilance, is of low to medium quality. Only sporadically were cases identified that scored high in all evaluated categories. On average, the lowest overall quality was found to be in cases reporting diazepam (mean 10.7±5.9, median 9), and fentanyl (mean 10.7±5.7, median 10), while the highest quality was observed in cases reporting paracetamol toxicity (median 13.6±5.8, median 14).

Cases reported from non-EEA countries were generally of lower quality compared to cases originating from EEA countries. The best quality was observed in cases reported by regulatory agencies.

Cases reported by non-HCPs (consumers) showed very low quality, which is expected, as reporting into a system such as Eudravigilance requires a high degree of background medical knowledge.

### Quality Categories and Quality Scores

Overall, the mean quality score for the 5 evaluated substances (total of 500 evaluated cases) was 11.57, which falls in the medium quality class.

Table 5 summarizes the descriptive statistic of overall quality category scores per substance, while Table 6 summarizes the mean and median values of the individual quality category scores per substance.

**Table 5:** Descriptive statistics for quality category scores per substance

	Paracetamol	Diazepam	Fentanyl	Fluoxetine	Quetiapine
N	100	100	100	100	100
Mean	13.6	10.7	10.7	11.2	11.6
SD	5.8	5.9	5.9	5.7	5.7
Median	14.0	9.0	10.0	10.5	9.0
Q1	9.0	6.0	6.0	6.7	7.0
Q3	17.0	15.2	14.0	15.2	15.2
IQR	8.00	9.25	8.00	8.50	8.25
Mean quality class	Medium	Medium	Medium	Medium	Medium
Lowest total quality score (% of cases)	3 (1)	1 (1)	2 (2)	2 (2)	3 (2)
Highest total quality score (% of cases)	25 (2)	24 (2)	25 (1)	24 (2)	25 (1)
N of cases in very low quality category (0–5)*	10	22	19	18	6
N of cases in very high quality category (21–25)*	18	8	8	8	12

*The percentage of and the absolute number are the same, as 100 records were included for each substance

**Table 6:** Number of cases per each individual quality category score for 5 substances

	Paracetamol (n of cases*)	Diazepam (n of cases*)	Fentanyl (n of cases*)	Fluoxetine (n of cases*)	Quetiapine (n of cases*)
Pharmaceutical form score 0	34	55	59	55	32
Pharmaceutical form score 1	66	45	41	45	68
Dose/dosing regimen score 0	68	80	87	72	74
Dose/dosing regimen score 1	32	20	13	28	26
Quality of data on substance score 0	13	28	39	23	26
Quality of data on substance score 1	42	37	32	47	46
Quality of data on substance score 2	17	18	16	12	9
Quality of data on substance score 3	28	17	13	18	19
Intoxication type score 0	34	45	48	35	25
Intoxication type score 1	66	55	52	65	75
Time to event score 0	70	74	86	80	82
Time to event score 1	30	26	14	20	18
Adequacy of reported AEs score 0	6	8	3	16	7
Adequacy of reported AEs score 1	94	92	97	84	93
Quality of data on AEs score 0	10	14	34	17	9
Quality of data on AEs score 1	35	49	36	36	52
Quality of data on AEs score 2	32	19	12	16	24
Quality of data on AEs score 3	23	18	18	31	15
Age/age group score 0	22	47	60	62	44
Age/age group score 1	88	53	40	38	56
Sex score 0	7	6	13	5	2
Sex score 1	93	94	87	95	98
Medical history score 0	68	95	91	94	92
Medical history score 1	32	5	9	6	8
Indication score 0	76	92	83	71	78
Indication score 1	24	8	17	29	22
Main suspect(s) reported score 0	37	63	42	59	64
Main suspect(s) reported score 1	63	37	58	41	36
Concomitant adequately reported score 0	46	68	44	59	65
Concomitant adequately reported score 1	54	32	56	41	35
Quality of data on concomitant score 0	46	60	43	58	65
Quality of data on concomitant score 1	34	16	36	26	21
Quality of data on concomitant score 2	20	24	21	16	14
Seriousness score 0	45	33	6	44	41
Seriousness score 1	55	67	94	66	59
Quality of additional data score 0	14	12	20	26	6
Quality of additional data score 1	19	42	30	13	20
Quality of additional data score 2	16	13	16	19	36
Quality of additional data score 3	11	6	12	16	9
Quality of additional data score 4	19	17	12	15	14
Quality of additional data score 5	21	10	10	11	15

*The percentage of and the absolute number are the same, as 100 records were included for each substance

There was a statistically significant difference in mean quality scores was observed between substances (ANOVA F(4, 495) = 4.51, *p* = 0.00139; Kruskal–Wallis H(4) = 18.88, *p* = 0.00083).

Post-hoc analysis using a Tukey HSD test revealed that paracetamol had significantly higher mean scores than diazepam (mean difference 2.98, 95% CI 0.736–5.224, *p* = 0.0028), while no other pairwise comparisons reached statistical significance.

The distribution of total scores was not uniform across the range, both overall and for each substance, which was tested using a Chi-square goodness-of-fit.

**Fig. 1:**
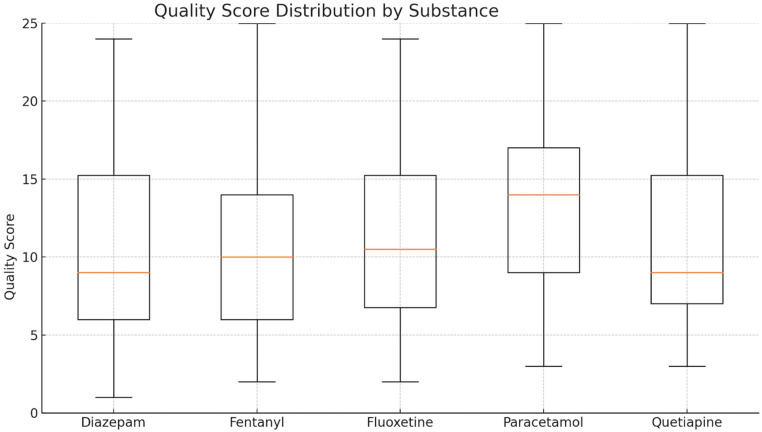
Distribution of total quality scores by substance. Boxplots showing the distribution of total quality scores for each substance, with median, interquartile range, and outliers. Paracetamol shows a higher mean score than Diazepam (*p* = 0.0028, Tukey HSD)

Residual normality was evaluated using the Shapiro–Wilk and D’Agostino–tests, both indicating significant deviation from normality. Q–Q plot inspection confirmed skew and heavy tails in the residual distribution. Figure 1 shows the quality score distribution by substance.

These findings suggest that, while overall quality is fairly similar for these substances, reports involving paracetamol tend to be more complete than those involving other substances. Although only the difference between paracetamol and diazepam reached statistical significance.

When scoring categories were evaluated individually, the results of the analysis showed a substantial variation in category performance, both across and within substances. Some categories, such as for example the adequacy of reported adverse events, had consistently higher scores, while others (for example indication for use of the suspect drug) were rarely reported.

Even substances with relatively high total scores showed low median scores in some categories, suggesting that a higher overall score can mask deficiencies in individual reporting components. Conversely, some substances with lower total scores achieved high scores in specific categories, reflecting focused strengths.

### Identified Quality Issues and Their Impact on the Overall Quality of the Report

According to the quality category scores, the most frequently encountered quality issue is the lack of information on the patient medical history and indication for the reported substance use (84.2% of cases), followed by the lack of information on time to event (78.4% of cases) and lack of information on the substance’s dose or dosing regimen (76.2% of cases). An overview of results is presented in Table 7.

**Table 7:** Most common quality issues and their impact on the overall quality of the case

Issue	% of reviewed cases with such issue	Impact on overall quality of report
Terms reported as adverse events are underlying diseases	7.8	High
Concomitant drugs or drugs used to treat the adverse event are reported as suspect	56.4	High
No data on patient medical history and/or indication for use	84.2	Medium
No data on dose/dosing regimen of suspect drug	76.2	Medium
No information of temporal relationship (time to event)	78.4	Low
No data on patient age and/or sex	25.8	Low

### Specific Important Quality Issues Identified for Individual Substances

#### Diazepam

Overall, the data quality was low. Several reports of toxicity with multiple drugs where 5 or more suspect drugs are listed, with no additional information.

In many cases it was noted that the additional information provided (e.g. narrative, literature reference) reported a different picture than the one seen in the case.

In 21% of cases, diazepam was not identified as the main suspect drug based on information in the narrative or publication, indicating a potential misclassification in the reported suspect drug designation.

#### Fentanyl

The majority of cases (85%) report a fatal outcome, therefore the reported PTs are usually very few and general (e.g. drug toxicity, intentional misuse…), as data in the initial report is usually scarce, and no follow-up is conducted for most of these cases. It is important to note that almost all cases report addiction and drug abuse, which is in line with the alarming situation that is developing with the opioid crisis worldwide [7]. During the review of cases of fentanyl toxicity, it was observed that in 32 out of 100 cases co-reported the abuse of an illegal drug (cocaine, heroin, methamphetamine), while almost all other cases co-reported abuse of prescription medications (oxycodone, tramadol, hydromorphone).

## Discussion

Eudravigilance database was established as a central repository for adverse event reports, which should be used for evaluating the benefits and risks of medicines during their development and monitoring their safety following their authorization in the European Economic Area (EEA). Although every Marketing Authorization Holder in the EEA is required to maintain an internal database of adverse events, data from Eudravigilance should help in the detection, and more importantly, validation of safety signals that arise during the postmarketing phase of a medicinal product.

To adequately evaluate such signals, it is of crucial importance that the data, extracted from Eudravigilance meets a certain degree of quality; if this is not the case, the results of analyses can be misleading, and can negatively affect the safety profile of the medicinal product in question. Additionally, the generation of irrelevant safety signals that are based on low-quality data, increases the workload in pharmacovigilance departments of Marketing Authorization Holders and regulators alike.

Our analysis randomly retrieved 500 reports of drug toxicity for five drugs that are most commonly implicated in accidental and intentional intoxications. Overall, the quality of the cases was relatively low, with mean quality category scores ranging from 10 to 13 out of 25. Especially two quality issues that were frequently encountered and have the potential to significantly impact the causality assessment: underlying conditions reported as adverse events, and concomitant drugs or drugs used to treat adverse events reported as suspect.

Additionally, since seriousness of a case is determined by assessing the seriousness of each reported adverse event, a wrong reporting can “upgrade” a case to a serious case, even if it is not. This in turn affects not only the quality of data, but also the timelines for reporting to other stakeholders, for example when such a case is obtained from Eudravigilance by means of ICSR download.

In the analysis of total quality scores, reports of paracetamol toxicity achieved higher mean scores compared to the other four evaluated substances, but statistical significance was reached only in comparison to reports of diazepam toxicity, which displayed the lowest total quality score. Distributional analysis showed that scores were not uniformly spread across quality categories, which likely reflects systematic reporting tendencies more than random variation. Category-level analyses revealed substantial variation in quality across reporting categories. Reports may excel in certain categories while underperforming in others, which indicates that reporting quality is multi-dimensional. Improving ICSR quality therefore requires both targeted (category-specific) improvements and a broader strategy to enhance overall completeness of reported information.

Many cases were identified where supporting/additional information clearly indicated which was the main drug that caused the event, but still irrelevant concomitant drugs are reported as suspect. If the reviewer does not perform a very thorough analysis (if only quantitative data are considered), the results of the analysis can be very wrong.

Nevertheless, there were several cases identified (over 20% of all evaluated cases) where the cited publication/background information reports much more information than what is actually included in the case. This could indicate that the person, who performed data entry into Eudravigilance, either did not have the required knowledge to adequately insert the case in the database or did not have the necessary time. In some cases, it is clearly evident that the reporter only inserted the basic necessary data for a case to be valid, not bothering to provide additional information that, when reviewed in further detail, painted a totally different picture of the event.

Another issue that arises from the evaluated reports is the applicability to registered medicinal products. For example, several cases were found to co-report fentanyl with heroin, cocaine and other illegal drugs of abuse, so it is plausible to assume that such cases are attributable to illegal fentanyl (produced by criminal organizations). It would therefore not be scientifically correct to “generalize” such findings to legal fentanyl-containing products (e.g. fentanyl patches) that are produced in a GMP-grade facilities.

Within Eudravigilance, there is the substance entry named FENTANYL (NON-PRESCRIPTION), which would, if reported correctly, enable a more adequate analysis of the safety of pharmaceutical grade, prescription fentanyl. Of the 100 evaluated cases, only one reported this substance as suspect, although most of them are clearly related to illicit fentanyl abuse, based on the context or based on additional information in the narrative or publication.

Overall, the quality of cases reported in Eudravigilance raises concerns for safety physicians that use these data to identify and assess safety signals. There are many pharmacovigilance processes in place that use this kind of data, including signal detection, benefit-risk analyses and periodic safety update reports. Despite being thoroughly described in the applicable guidelines, such as GVP, there is no comprehensive guidance provided that would warn the evaluators of this problem.

Our review clearly indicates that, in order to keep Eudravigilance as a valid data source, actions are needed to improve data quality, and these actions should be implemented by regulators and Marketing Authorization Holders alike.

In our opinion, the most important call to action is an overall mindset change of all stakeholders, involved in case reporting. Reporters should consider how their submissions will be interpreted by someone without prior knowledge of the case or access to supporting documentation. 

This could be achieved by strengthening the cooperation between pharmacovigilance and medical departments, for example with regular trainings for data entry responsible persons, focusing on quality of reporting. Other possible approaches are to strengthen the quality control process, which should involve a medically trained person (this is especially important for cases, reported by consumers), promote the request of follow-up information whenever possible and to optimize the signal detection processes, which should consider data quality.

Our study has several limitations. The most important is the fact that the scoring is partially dependent on the subjective assessment by one physician only. The scoring system was developed to be as objective as possible, but since the assessed data are not fully standardizable (e.g., case narrative or literature publication from where the ICSR is reported), an element of subjectivity is always present. Nevertheless, this is the first application of the scoring system, and further work will be pursued to reduce as much as possible scoring based on subjective judgement and to validate the system on larger samples and multiple scorers.

In conclusion, a review of data quality of cases, reported in Eudravigilance, showed that there is plenty of room for improvement. The cases are very often reported with very basic errors (such as reporting underlying conditions or indications for use as adverse events), which creates confusion during medical assessment and can result in incorrect signal detection. In future, efforts to improve reporting of adverse events should be focused not only on quantitative compliance, but also on data quality and quality control.

It is the responsibility of us all that work in pharmacovigilance to share the highest possible level of data quality within public databases and thus enabling an adequate safety overview of all medicinal products that are available to our patients.

## Data Availability

The data supporting the findings of this study are available from the author, however access is restricted, as the data were obtained from Eudravigilance granted by the European Medicines Agency. Data are located in controlled access data storage and should be fully anonymized prior to sharing.

## Footnotes

1 TCA overdose accounted for 1.12 exposures per 10,000 population in 1992. Recently, the trend for antidepressant overdose has shifted more towards selective serotonin reuptake inhibitors (SSRIs) [5].
